# Preference for Face‐to‐Face Contraceptive Service Delivery Post‐COVID‐19 Pandemic: A Cross‐Sectional Study

**DOI:** 10.1111/1471-0528.18323

**Published:** 2025-08-11

**Authors:** Sophie Patterson, Nicola Rennie, Sue Mann, Ona McCarthy, Melissa Palmer, Rebecca S. French

**Affiliations:** ^1^ Lancaster Medical School Lancaster University Lancaster UK; ^2^ Homerton Healthcare NHS Foundation Trust London UK; ^3^ Department of Public Health, Environments and Society London School of Hygiene and Tropical Medicine London UK

**Keywords:** contraception, digital health, health inequities, public health, reproductive health

## Abstract

**Objective:**

To measure the prevalence of, and social positions associated with, preference for solely face‐to‐face contraceptive service delivery among women and people assigned female at birth in post‐COVID‐19 pandemic England.

**Design:**

Cross‐sectional online study.

**Setting:**

England.

**Sample:**

The Reproductive Health Survey for England (RHSE) recruited women and people assigned female at birth aged 16–55 living in England using an online non‐probability convenience sampling strategy from September–October 2023. The study population was limited to contraception users who answered the question of interest.

**Methods:**

Multivariable logistic regression identified variables independently associated with preference for face‐to‐face services.

**Main Outcome Measures:**

Preference for face‐to‐face services, derived from response to the question ‘How would you prefer to access contraceptive services?’ (face‐to‐face vs. telephone/video/website/combination/no preference).

**Results:**

The study population included 28 328 participants: median age was 30 (IQR:24–38), 92.5% (*n* = 26 193) reported White ethnicity, and 96% (*n* = 27 296) identified as a woman/girl. Preference for solely face‐to‐face services services was reported by 24.7% (*n* = 6992/28 328). In adjusted analysis, preference for face‐to‐face was associated with younger and older age; not having degree‐level qualifications, self‐reporting financial hardship, living with a disability, identifying as a woman/girl, and not being in a (cohabiting) relationship. Whilst there was a significant independent association between paid employment and preference for face‐to‐face services, effect direction was dependent on ethnicity.

**Conclusions:**

Although a minority of participants reported a preference for solely face‐to‐face services, they may represent those with the highest unmet need for contraceptive services. Maintaining choice within contraceptive service delivery in an increasingly digitised healthcare landscape is crucial to advance equitable, person‐centred reproductive healthcare.

## 1 Introduction

The past 5 years have brought a shift in the way contraceptive services are delivered. In releasing its first digital health guidelines in 2019, the World Health Organisation (WHO) endorsed the incorporation of digital technologies to complement and enhance health systems, with the aim of expanding access to equitable and person‐centred care [1]. In the same year, WHO's guidelines on self‐care interventions for sexual and reproductive health (SRH) framed the digital health movement as an enabler of self‐care, transitioning the promotion and maintenance of SRH beyond traditionally clinical settings to advance empowerment, autonomy and agency [2].

The COVID‐19 pandemic accelerated the expansion of remote (telephone, video or online) consultations on an international scale, with social distancing restrictions triggering an urgent necessity to relocate healthcare interactions beyond traditional face‐to‐face clinical settings [3]. In England, this shift was mirrored in reproductive healthcare, whereby novel access was granted to early medical abortion care via telemedicine [4] along with an upscaling of remote contraceptive services [5, 6].

Digital infrastructure investment and refinement of protocols to safely and effectively deliver remote consultations during the COVID‐19 pandemic opened the door for sustained remote consultations in SRH [3]. Remote consultations have been characterised as an efficient way to streamline, and respond to an increasing demand for, pressured and underfunded SRH services [5, 6]. By responding to lived realities (flexibly accommodating caring responsibilities and work schedules, and overcoming geographical barriers [5, 7, 8]), remote services have the potential to advance gender equity, promote empowerment, and optimise SRH [9, 10]. However, this modality shift may not be welcomed by all. Stakeholders have voiced concerns about potential inequity in access to, and benefit from, remote contraceptive services [5, 11].

In response to calls from previous work [5, 12], the aim of this study was to better understand *preferences* for mode of contraceptive service delivery in a post‐COVID‐19 landscape, and to consider implications for reproductive health equity. To this end, our objectives were to use a cross‐sectional survey to measure the prevalence of, and social positions associated with, self‐reported preference for solely face‐to‐face contraceptive services among women and people assigned female at birth. This scholarship builds upon earlier work [7, 13, 14, 15] examining uptake and experiences of remote contraceptive services during the COVID‐19 pandemic to consider preferences for contraceptive service delivery beyond the influence of pandemic‐related factors.

## 2 Methods

### 2.1 Study Setting

This study was conducted in England. At the time of the 2021 Census, the female population of England and Wales was 30.4 million (51% of the total population) [16]. Approximately half (15.2 million) of the female population was aged 16–54, and among this age demographic 14.3 million (94%) reported that their gender identity was the same as that registered at birth [17].

Almost half of pregnancies in Britain are unplanned [18] and the national abortion rate continues to climb [19]. Women's Health was acknowledged as a national priority with the release of The Department of Health and Social Care's (DHSC) Women's Health Strategy in 2022, which included ambitions to increase access to contraception and expand the use of digital technologies [20].

### 2.2 Data Source

Data were drawn from the Reproductive Health Survey for England (RHSE); a cross‐sectional digital survey, commissioned by DHSC to better understand reproductive health and experiences with care among women and people assigned female at birth living in England [21, 22]. RHSE used an online non‐probability convenience sampling strategy to recruit people aged 16–55 who were resident in England and assigned female at birth (inclusive of people who identified as a woman/girl, man/boy, non‐binary, trans, or thought of themselves ‘in another way’). A detailed description of the survey and sampling strategy appears in previous publications [21, 22]. In brief, the sampling strategy aimed to rapidly and conveniently recruit a sample broadly reflective (but not representative) of the English population, in terms of age, ethnicity, education level and region of residence. The digital marketing strategy and recruitment materials were tailored to increase recruitment among groups (minority ethnic groups, young adults and those without a degree) underrepresented in a pilot survey carried out in 2021 [21] (e.g., through adapting survey advertisements and promotional materials and conducting outreach through partner organisations). Recruitment took place for 6 weeks between September–October 2023 via social media (Facebook and Instagram), press coverage and network dissemination. During the recruitment phase, the sample was monitored weekly, with additional resources targeted towards demographic groups underrepresented based on comparison with 2021 Census (e.g., through targeted adverts on Facebook and Instagram) [22].

Participants self‐completed an online survey in English using Snap Survey. The survey included questions about periods and menopause; preventing and planning pregnancy; pregnancy experiences; and reproductive health conditions. No incentives were used to encourage survey participation.

### 2.3 Patient Involvement

The RHSE was reviewed and tested by a lived experience group, with feedback incorporated into the final survey. A separate patient and public involvement discussion group was conducted specifically to refine survey questions related to preferences for the modality of contraceptive service delivery. Additionally, a stakeholder group was convened including representatives from community organisations serving women and those assigned female at birth experiencing various forms of inequity in Northern England to discuss study findings and recommendations related to this analysis.

### 2.4 Theoretical Framework

Intersectionality recognises that women are not heterogeneous, and their varied and multiple social positions overlap (rather than existing on a single axis) to shape advantage or disadvantage, which can drive health inequities [23, 24, 25]. Considering digital health‐related outcomes through an intersectional lens is important, as they are shaped not only by gender, but also by social positions (e.g., age, class, physical ability, geographical location) [9]. This study considered the influence of varied and multiple social positions to examine how they may jointly shape preferences for modality of contraceptive service delivery, acknowledging the complexity and diversity of lived experience.

### 2.5 Ethical Considerations

Ethical approval for the RHSE was granted by the Observational/Interventions Research Ethics Committee at the London School of Hygiene and Tropical Medicine. Further ethical approval related specifically to this study was granted by the Faculty of Health and Medicine Research Ethics Committee at the University of Lancaster. Prior to completing the online survey, participants were required to read an online information sheet about the RHSE and to provide voluntary informed consent via completion of an online form.

### 2.6 Inclusion Criteria

We limited inclusion to participants who answered the survey question of interest. Due to the survey design and filter questions used, participants reporting the use of no contraceptive method, tubal ligation, partner vasectomy, lactational amenorrhoea method, safe period/calendar method/rhythm method, withdrawal, fertility awareness apps, or avoidance of penetrative sex were not asked the question exploring preferences for mode of contraceptive service delivery, so could not be included in this analysis.

## 3 Measures

### 3.1 Main Outcome

The main outcome was preference for solely face‐to‐face contraceptive service delivery, measured by response to the question: ‘How would you prefer to access contraceptive services?’. Participants were asked to select one of the following responses: solely face‐to‐face; telephone consultation; video consultation; health service website; combination of online/video/telephone and face‐to‐face services; or no preference. Responses were dichotomized as ‘preference for solely face‐to‐face’ vs. ‘no preference for solely face‐to‐face’ (including all other response options). This grouping was chosen based on its relevance for healthcare resourcing and health equity given ambitions for a digital shift in the delivery of health services [1, 26].

### 3.2 Covariates

Consistent with an intersectionality framework, we included variables reflecting social positions hypothesised to influence preference for face‐to‐face services based on a priori knowledge, existing literature and availability within the dataset. Sociodemographic characteristics included: age group (16–29 vs. 30–39 vs. 40–55); whether participants were born in the UK; ethnicity (consistent with broad 2021 Census categories [27]); region of residence (derived from postcode data and presented across seven regions of England's National Health Service [NHS]); and gender identity. In the survey, participants were given the option to select one or more of the following gender identity categories: woman/girl, man/boy, non‐binary, trans, and ‘think of myself in another way’. For this analysis, we dichotomised the gender identity variable as follows: woman/girl exclusively vs., man/boy, non‐binary, trans, those who thought of themselves ‘in another way’, or reported a combination of responses. Socioeconomic variables included: education level (degree or equivalent qualification), current paid employment, index of multiple deprivation (IMD) quintile (derived from postcode) and self‐reported financial hardship. Financial hardship was measured by response to the question ‘How well would you say you yourself are managing financially these days?’ and assessed across four levels (living comfortably vs. doing alright vs. just getting by vs. finding it difficult). This variable has been used in previous analyses and is included as a question in the UK Household Longitudinal Study [28, 29].

Health‐related variables included: self‐reported health status (assessed across three levels: good vs. fair vs. bad), and presence of disability (defined in accordance with the 2021 Census as presence of long‐term physical or mental health conditions that limit day‐to‐day activities [27]). Family status variables included: relationship status (assessed across four levels: in a relationship living together vs. in a relationship not living together vs. not in a relationship vs. other/PNTA) and experience of parenthood (measured as history of live birth and/or parent to a child they did not give birth to).

### 3.3 Statistical Analysis

Descriptive statistics were used to characterise the distribution of the variables under study (count and percentage for categorical variables and median and interquartile range [IQR] for continuous variables). Sociodemographic and health‐related variables were compared between participants self‐reporting a preference solely for face‐to‐face services and those preferring other/combined modes of delivery, using Pearson's chi‐squared test (Fisher's exact for counts < 5) for categorical variables, and Wilcoxon test for continuous variables due to non‐normality.

Multivariable logistic regression identified factors independently associated with preference solely for face‐to‐face contraceptive services. Variables were pre‐specified for inclusion in the model based on literature review [5, 7, 8, 11, 12, 13, 14, 15, 30, 31, 32, 33, 34, 35], and interactions between these variables were explored through visualisation of interaction plots. Interactions that were identified (e.g., preference for face‐to‐face services vs. ethnicity by paid employment) were considered within the model to examine how multiple social positions may jointly (rather than independently) shape outcomes [25, 36, 37]. Where two variables that measured similar phenomena (e.g., health and disability) exhibited high collinearity, and therefore could not both be included in the model, the variable deemed most relevant based on literature review [38, 39] was selected.

Each variable was examined to determine the percentage of missing data, whether it was missing completely at random, and to consider whether missing values were predictive. Missing responses were imputed for variables with missing data < 5% (including gender identity, born in the UK, ethnicity, degree or equivalent qualification, financial hardship, disability and relationship status) to preserve statistical power. The multivariate imputation using chained equations (MICE) method was used to perform the imputation, with all variables included as predictors for the imputation models to minimise bias [40]. The imputation approach used a combination of logistic regression, polytomous regression, and proportional odds models to impute missing values. No major inconsistencies were found when comparing model fit for imputed and non‐imputed data. If variables had a large proportion of missing data (e.g., ‘NHS region’ with > 10% missing data) they were not a candidate for imputation, and two scenarios were considered: excluding the variable from the model or including missing data as a category in the model. Given that the ‘NHS region’ variable was derived from postcode data, we also considered the possibility that the missing category could be a latent variable (e.g., non‐response could be associated with residence in a deprived region). As such, we decided to include missing in the model as a response option for this variable.

All analyses were conducted using R statistical software, version 4.4.1, ‘Race for your Life’ [41]. *p*‐values were two‐sided and considered statistically significant at *α* = 0.05.

### 3.4 Sub‐Analysis

The survey question of interest (‘How would you prefer to access contraceptive services?’) was worded hypothetically and not specifically related to the participant's current contraceptive method. However, participant preference may be influenced by current contraceptive circumstances. For example, long‐acting reversible contraceptive (LARC) methods (including implant, injection, hormonal intrauterine system or copper coil/intrauterine device) [42, 43] would necessitate a face‐to‐face consultation at some point during the care journey. Therefore, the selected model was also tested among a sub‐group of participants who reported using non‐LARC methods (including male/female condoms, cap/diaphragm, combined oral contraceptive pill, progesterone only pill, vaginal ring, contraceptive patch, emergency contraceptive pill, spermicides, other method of contraception), who would not typically *require* a face‐to‐face consultation for fitting/insertion of their contraceptive method.

### 3.5 Sensitivity Analysis

We were aware that the online recruitment strategy and online survey completion were likely to influence sample diversity and the nature of participant responses; therefore, we planned in advance of this analysis to crudely test the main findings among a small offline sample. Purposive recruitment sampled social groups underrepresented in the main study population. Participants were recruited through community organisations in the North West, including a food bank and community organisations serving women and people assigned female at birth experiencing various forms of inequity. Participants completed a short‐form paper‐based version of the RHSE. Sociodemographic characteristics and preference for face‐to‐face contraceptive services were compared between the main study population and the offline sample using Pearson's chi‐squared test (Fisher's exact for counts < 5). Univariable relationships between the outcome variable and key variables identified in the main study population were tested among the offline sample.

## 4 Results

Among 59 332 participants who completed the RHSE, 28 328 (48%) were included in this study. Characteristics of the study population are shown in Table 1. The median participant age was 30 years (interquartile range [IQR]: 24–38) and 96% (*n* = 27 296) identified as a woman/girl. Among this study population of contraceptive users, most (62%, *n* = 17 864) used a non‐LARC method.

**TABLE 1:** Characteristics of study population (*n* = 28 328).

Characteristic	Median (IQR); *n* (%)
**Age group**	
16–29	13 538 (48)
30–39	8365 (30)
40–55	6425 (23)
**Gender identity**	
Woman/girl	27 296 (96.4)
Man/boy, non‐binary, trans, or thinks of themselves ‘in another way’	1024 (3.6)
Missing	8 (< 0.1)
**Born in the UK**	
Yes	25 413 (89.7)
No	2815 (9.9)
Missing	100 (0.4)
**Ethnicity**	
White	26 193 (92.5)
Mixed or multiple ethnic groups	982 (3.5)
Asian or Asian British	565 (2.0)
Black, Black British, Caribbean or African	302 (1.1)
Other ethnic group	129 (0.5)
Missing	157 (0.6)
**Degree or equivalent qualification**	
Yes	20 817 (73.5)
No	7331 (25.9)
Missing	180 (0.6)
**In paid employment**	
Yes	23 170 (81.8)
No	5158 (18.2)
Missing	0 (0.0)
**Self‐reported financial hardship**	
Living comfortably	5819 (20.5)
Doing alright	12 686 (44.8)
Just getting by	6741 (23.8)
Finding it difficult	3030 (10.7)
Missing	52 (0.2)
**NHS Region**	
London	4715 (16.6)
South East	4114 (14.5)
South West	3179 (11.2)
East of England	2597 (9.2)
Midlands	4077 (14.4)
North East and Yorkshire	3579 (12.6)
North West	2828 (10.0)
Missing	3239 (11.4)
**Index of Multiple Deprivation (IMD) quintile**	
1	3215 (11.3)
2	5176 (18.3)
3	5547 (19.6)
4	5604 (19.8)
5	5547 (19.6)
Missing	3239 (11.4)
**Self‐reported health status**	
Good	19 505 (68.9)
Fair	6084 (24.2)
Bad	1882 (6.6)
Missing	77 (0.3)
**Disability**	
Yes	10 425 (22.3)
No	17 744 (62.6)
Missing	159 (0.6)
**Relationship status**	
In a relationship, living together	16 826 (59.4)
In a relationship, not living together	5692 (20.1)
Not in a relationship	5182 (18.3)
Other, PNTA	445 (1.6)
Missing	183 (0.6)
**Experience of parenthood**	
Yes	18 558 (66)
No	9299 (33)
Missing	471 (1.7)

Abbreviations: IMD, index of multiple deprivation; PNTA, prefer not to answer.

### 4.1 Preference for Modality of Contraceptive Service Delivery

Hybrid (combination of remote and face‐to‐face) delivery was most frequently reported (38%) as the preferred modality of contraceptive service delivery, with 19% reporting no preference. Among those reporting a preference for remote contraceptive services (18%), health service website was the most prevalent response (Table 2).

**TABLE 2:** Preferred mode of contraceptive service delivery among study population (*n* = 28 328).

Mode of contraceptive service delivery	*N* (%)
Solely face‐to‐face consultation	6992 (24.7)
Combination of remote (online/video/telephone) and face‐to‐face services	10 888 (38.4)
No preference	5241 (18.5)
Health service website	3267 (11.5)
Telephone contraceptive consultation	1617 (5.7)
Video consultation	323 (1.1)

A quarter (25%, *n* = 6992) of participants reported a preference for solely face‐to‐face contraceptive services. In unadjusted analysis (Table 3), compared to those reporting a preference for remote or hybrid services or no preference, participants reporting a preference for solely face‐to‐face contraceptive services were more likely to be younger (16–29 vs. 30–39: odds ratio [OR]:1.48, 95% confidence interval [CI]: 1.38–1.58) or older (40–55 vs. 30–39; OR: 1.66, 95% CI: 1.54–1.79); not have a university‐level degree or equivalent qualification (OR 1.44, 95% CI: 1.36–1.53), not be in paid employment (OR: 1.30, 95% CI: 1.22–1.39); report financial hardship (finding it difficult vs. living comfortably; OR:1.44, 95% CI: 1.31–1.60); live in certain regions outside London (e.g., North East and Yorkshire vs. London, OR: 1.30, 95% CI: 1.18–1.44); report a disability (OR 1.16, 96% CI: 1.10–1.23); and not be in a (co‐habiting) relationship (e.g., in a relationship not living together vs. in a co‐habiting relationship; OR: 1.32, 95% CI: 1.23–1.41). Participants not born in the UK (vs. born in the UK, OR: 0.91, 95% CI 0.83–0.99) and those reporting Asian or Asian British ethnicity (vs. White; OR: 0.76, 95% CI: 0.62–0.93) were less likely to report a preference for solely face‐to‐face contraceptive services.

**TABLE 3:** Odds ratios for correlates of preference for solely face‐to‐face contraceptive services among study population (*n* = 28 328).

Characteristic	Number of participants	Percentage reporting preference for solely face‐to‐face services	Unadjusted OR (95% CI)	Adjusted OR (95% CI)
**Age group**				
30–39	8365	19.3	1.00	1.00
16–29	13 538	26.2	1.48 (1.38–1.58)	1.26 (1.17–1.35)
40–55	6425	28.5	1.66 (1.54–1.79)	1.65 (1.52–1.78)
**Gender identity**				
Woman/girl	27 303	24.7	1.00	1.00
Man/boy, non‐binary, trans, or thinks of themselves ‘in another way’	1025	24.2	0.97 (0.84–1.12)	0.85 (0.73–0.99)
**Born in the UK**				
Yes	25 504	24.9	1.00	1.00
No	2824	23.0	0.91 (0.83–0.99)	1.01 (0.91–1.11)
**Ethnicity**				
White	26 343	24.8	1.00	1.00
Asian or Asian British	566	20.0	0.76 (0.62–0.93)	0.85 (0.67–1.08)
Black, Black British, Caribbean or African	306	23.5	0.94 (0.72–1.22)	1.15 (0.86–1.52)
Mixed or multiple ethnic groups	984	23.2	0.92 (0.79–1.06)	1.01 (0.84–1.19)
Other ethnic group	129	26.4	1.08 (0.72–1.59)	1.35 (0.84–2.09)
**Degree or equivalent qualification**				
Yes	20 942	22.8	1.00	1.00
No	7386	30.0	1.44 (1.36–1.53)	1.28 (1.20–1.36)
**In paid employment**				
Yes	23 170	23.8	1.00	1.00
No	5158	28.9	1.30 (1.22–1.39)	1.17 (1.09–1.26)
**Financially managing**				
Living comfortably	5836	21.8	1.00	1.00
Doing alright	12 721	24.1	1.14 (1.06–1.23)	1.10 (1.02–1.19)
Just getting by	6741	26.6	1.30 (1.20–1.41)	1.17 (1.08–1.28)
Finding it difficult	3030	28.6	1.44 (1.31–1.60)	1.23 (1.11–1.37)
**NHS Region**				
London	4715	21.6	1.00	1.00
South East	4114	24.8	1.20 (1.08–1.32)	1.06 (0.96–1.18)
South West	3179	26.3	1.29 (1.16–1.44)	1.15 (1.04–1.29)
East of England	2597	25.2	1.22 (1.09–1.36)	1.07 (0.95–1.20)
Midlands	4077	26.0	1.27 (1.15–1.40)	1.12 (1.01–1.24)
North East and Yorkshire	3579	26.4	1.30 (1.18–1.44)	1.17 (1.06–1.30)
North West	2828	24.6	1.18 (1.06–1.32)	1.07 (0.96–1.20)
Missing	3239	23.4	1.11 (1.00–1.23)	1.01 (0.90–1.12)
**Disability**				
No	17 846	23.6	1.00	1.00
Yes	10 482	26.5	1.16 (1.10–1.23)	1.07 (1.01–1.14)
**Relationship status**				
In a relationship, living together	16 950	23.0	1.00	1.00
In a relationship, not living together	5720	28.2	1.32 (1.23–1.41)	1.20 (1.11–1.29)
Not in a relationship	5210	26.5	1.21 (1.13–1.30)	1.12 (1.04–1.21)
Other, PNTA	448	21.9	0.94 (0.75–1.18)	0.88 (0.70–1.11)
Ethnicity*In paid employment				
White * Yes	21 606	23.9	—	1.00
Asian or Asian British*No	113	21.2	—	0.79 (0.46–1.30)
Black, Black British, Caribbean or African*No	52	11.5	—	0.29 (0.11–0.67)
Mixed or multiple ethnic groups*No	224	23.2	—	0.73 (0.51–1.05)
Other ethnic group*No	32	21.9	—	0.60 (0.22–1.49)

Abbreviation: PNTA, prefer not to answer.

In the adjusted logistic regression model (Table 3), characteristics independently associated with a preference for solely face‐to‐face contraceptive services included older (40–55 vs. 30–39; OR 1.65, 95% CI: 1.52–1.78) or younger (16–29 vs. 30–39; OR: 1.26, 95% CI: 1.17–1.35) age; no degree or equivalent qualification (OR: 1.28, 95% CI: 1.20–1.36); financial hardship (e.g., finding it difficult vs. living comfortably, OR: 1.23, 95% CI: 1.11–1.37); living with a disability (OR: 1.07, 95% CI: 1.01–1.14); and not being in a (cohabiting) relationship (e.g., in a relationship not living together vs. in a co‐habiting relationship; OR: 1.20, 95% CI: 1.11–1.29). Compared to participants identifying as a woman/girl, those reporting diverse gender identities (including identifying as man/boy, non‐binary, trans or who thought of themselves ‘in another way’) had reduced odds of preferring solely face‐to‐face services (OR: 0.85 95% CI: 0.73–0.99). Regional differences also emerged, with participants living in the South West (OR: 1.15; 95% CI: 1.04–1.29), Midlands [OR: 1.12; 95% CI: 1.01–1.24] or North East and Yorkshire [OR: 1.17; 95% CI: 1.06–1.30] more likely to prefer face‐to‐face services than those living in London.

For participants reporting White ethnicity, those not in paid employment were significantly more likely to report a preference for solely face‐to‐face services compared to those in paid employment. Conversely, participants reporting Black British, Caribbean or African ethnicity in combination with no paid employment had significantly lower odds of reporting a preference for solely face‐to‐face services compared to White participants in paid employment.

### 4.2 Sub‐Analysis

Among non‐LARC users, 17% (*n* = 3084) reported a preference for solely face‐to‐face contraceptive services (Table S1). When model reliability was tested among non‐LARC users, directions of associations were broadly consistent with the main study population; however, some variables (e.g., gender identity, disability, regional differences) lost significance, likely due to reduced power in the smaller sample (Table S2).

### 4.3 Sensitivity Analysis

For the sensitivity analysis, 76 participants were recruited offline to complete the short‐form paper‐based RHSE. Compared to the main study population, a greater proportion of offline participants were older (median age 38 [IQR 32–40] vs. 30 [IQR 24–38]), non‐White ethnicity (20% vs. 7%), without degree‐level qualification (58% vs. 26%), residing in the most deprived IMD quintile (46% vs. 20%), not in paid employment (66% vs. 18%), finding it difficult financially (25% vs. 11%), not in a relationship (49% vs. 18%), and were living with a disability (61% vs. 22%) (all *p* < 0.001). These observations held true when restricting the offline sample to contraceptive users (*n* = 36).

In terms of preferred mode of contraceptive delivery, a lower proportion of offline participants reported ‘no preference’ compared to the main sample (5% vs. 19%, *p* < 0.001, respectively, Table S3). Offline participants were significantly more likely to prefer solely face‐to‐face services compared to the main study population (47% vs. 25% *p* < 0.001, respectively—with findings consistent when limited to offline participants using contraception). As the offline sample overrepresented social positions associated with preference for face‐to‐face services in the main sample, this aligned with our findings. In unadjusted analysis, the direction of effect for key social positions (including paid employment, educational attainment, disability, and financial hardship) was broadly consistent with the main analysis (Table S4).

## 5 Discussion

### 5.1 Main Findings

Hybrid services emerged as the preferred modality of contraceptive service delivery in this digitally connected study population. Although a minority of participants reported a preference for solely face‐to‐face services, they may represent those with the highest unmet need for contraceptive services. Compared to participants reporting a preference for remote or hybrid services (or no preference), preference for solely face‐to‐face services was independently associated with disadvantaged social positions (including financial hardship, lower educational attainment, and living with a disability), identifying as a woman/girl, younger or older age, and not being in a (cohabiting) relationship. Regional differences also emerged, with participants living in the South West, Midlands, or North East and Yorkshire more likely to prefer face‐to‐face services compared to those living in London. Whilst there was a significant independent association between paid employment and preference for face‐to‐face services, the effect direction was dependent on ethnicity.

### 5.2 Strengths and Limitations

A key limitation of this study was the digital recruitment and online survey completion, meaning that digitally excluded groups and those preferring not to engage with digital platforms were underrepresented. We crudely tested findings among an offline sample; however, the modest number of participants limited the scope of this sensitivity analysis.

A strength of this study was the large sample size. However, we are unable to claim that the prevalence estimates are representative of the wider English population given the non‐probability convenience approach to sampling [22]. When compared to the 2021 Census, the RHSE overrepresents individuals with a degree‐level qualification, White ethnicity, and living in less deprived regions [22]. The overrepresentation of people occupying privileged social positions in our sample may underestimate the preference for solely face‐to‐face contraceptive services compared to the general population.

Another strength of the RHSE is that it includes the perspectives of trans and gender diverse individuals. However, more targeted work is needed to understand preferences and experiences across minoritised gender identities. The range of social and reproductive health indicators collected enabled measurement of preferences across a range of social positions. Whilst this study applied an intersectional lens, the composition of the study population limited analyses of intersecting inequity among certain groups (e.g., minoritised ethnic groups). Data limitations precluded clarification of current living circumstances. Specifically, whilst the survey collected information on previous live births, it did not assess whether the children were still living, or whether they were cohabiting with the participant; therefore, it was not possible to ascertain current family status and household composition/caring responsibilities, which may impact preferences for mode of contraceptive service delivery [5]. Furthermore, the survey instrument was in English, representing a barrier to participation for those with limited English language proficiency, who additionally face barriers to engagement with remote health services [44].

This research was conducted in England; thus, findings may not be directly transferrable to other international settings with different populations and digital health infrastructures. However, given the international appetite to embrace digital health innovations [1], this work presents important conclusions related to preferences for solely face‐to‐face contraceptive services among people experiencing socioeconomic disadvantage in an increasingly digitised post‐COVID healthcare landscape.

### 5.3 Interpretation

To our knowledge, this study represents the broadest examination of preferences for contraceptive service delivery in post‐COVID‐19 pandemic England, with a majority of participants preferring hybrid modalities. Our findings broadly align with a study of patient‐initiated demand across 154 General Practices in England, in which a minority of patient requests stated a preference for face‐to‐face consultations post COVID‐19 pandemic, with a decline in preference for face‐to‐face consultations observed since the onset of the COVID‐19 pandemic [45]. For remote contraceptive services specifically, the literature supports good acceptability and uptake in England [45, 46] and internationally [47, 48]. However, acceptability and uptake may not correlate with preference; an important indicator of patient‐centred care [49]. Importantly, the NHS has committed to valuing patient choice in healthcare planning and delivery [50].

A notable proportion of participants in our study population reported no preference for mode of contraceptive service delivery, which may be explained by the increased familiarity and acceptability of remote and hybrid modes of delivery since 2019/2020 [45]. However, this finding may also reflect the sociodemographic composition of our sample, potentially with reduced barriers to accessibility and acceptability across the modalities of service delivery. Indeed, among the offline sample (with a higher prevalence of social disadvantage), the proportion of participants reporting no preference for mode of contraceptive service delivery was lower.

Whilst a minority (25%) of study participants preferred solely face‐to‐face contraceptive services, participants occupying disadvantaged social positions were more likely to report this preference. This finding must be contextualised by evidence that socioeconomically deprived groups are overrepresented among those with unmet contraceptive need in national [18, 22, 51] and international [52] contexts. Notably, an analysis using RHSE data found that participants who were ‘finding it very difficult’ financially had almost five times greater odds of an unplanned pregnancy in the previous year compared to those living comfortably [22]. Given that our study population is necessarily digitally connected, our findings may reflect broader medical or social needs that are less suited to a remote consultation [8, 11]. Whilst good uptake of remote contraceptive services has been demonstrated among socioeconomically deprived populations in London [46] other analyses among general medical patients suggest that people living in deprived regions are less likely to engage with digital health innovations [31, 53].

Trans and gender diverse participants had reduced odds of reporting a preference for face‐to‐face services. Our findings align with previous publications identifying remote delivery of health services as a means of reducing social and structural barriers to healthcare engagement for trans and gender diverse people [33, 34]. Although more work is needed to ensure appropriate recognition and accommodation of gender diversity in remote health service delivery [35]. In our study population, older and younger participants were more likely to report a preference for face‐to‐face services. Previous research among young people in UK [15, 54] and internationally [8, 55, 56] has identified barriers to engaging with remote SRH services, including low confidence or embarrassment advocating for SRH remotely, limited privacy in shared accommodation, and data security concerns. For older people, preferences for face‐to‐face services may reflect a desire for more holistic person‐centred care during menopause [57], which remote services may be less suited to delivering [8]. Qualitative research suggests poorer experiences of remote contraceptive consultations during the menopause [7], and a preference for face‐to‐face contact for initial menopause‐related consultations [58]. Whilst reduced digital literacy among older people (particularly women) is also an important consideration [59], this may be less relevant in our digitally recruited sample.

In this study, the direction of association between paid employment and preference for face‐to‐face services varied according to ethnicity. A potential explanation is that, compared to White counterparts, people of Black British, Caribbean or African ethnicity are more likely to work in caring and service occupations [60], requiring in‐person attendance and potentially offering less flexibility than other roles to schedule a remote consultation during working hours. Previous English studies have identified reduced uptake of remote primary care services among minoritised ethnic groups [31] and reduced likelihood of repeated engagement with remote contraceptive services [30]. Similarly, research conducted in the US identified ethnic disparities in quality of remote contraceptive care [12]. It is important to acknowledge that an intersection between class and racism may underlie our findings, negatively shaping experiences of in‐person healthcare interactions for participants living with overlapping systems of inequity. This work supports the importance of an intersectional lens to inform person‐centred contraceptive service delivery [9].

London was an outlier in this study, with residents less likely to prefer solely face‐to‐face contraceptive services compared to some other regions. Geographical disparities in remote contraceptive service uptake have been similarly observed in the US [12]. Inequitable implementation of digital health innovations may originate at individual, provider, and health organisational levels [61]. In England, access to free‐at‐point‐of‐delivery online contraceptive services varies due to budgetary constraints at Local Authority level, driving geographical inequities in choice of contraceptive service delivery [62]. Further contributing to an enabling remote health environment, London boasts the highest proportion of internet users nationally [59]. The internet has become an enabler of global healthcare access, whilst simultaneously introducing new challenges for health equity [8, 63, 64]. Smartphone ownership and internet access continue to rise; however, pockets of society remain digitally excluded. In 2018, 10% of UK adults were internet non‐users, with women, older people, people with disabilities, and economically inactive disproportionately represented [39]. This digital divide is even more stark globally [65].

## 6 Conclusion

Digital health technologies have been heralded as a means of improving socioeconomic equity in women's health [10]. Among participants of a national online reproductive health survey including women and those assigned female at birth, preference for solely face‐to‐face contraceptive services was reported by a quarter of participants and was independently associated with reporting disadvantaged social positions (including financial hardship, lower educational attainment and disability). Although a minority of participants reported a preference for solely face‐to‐face services, they may represent those with the highest unmet need for contraceptive services. Intersectional qualitative scholarship is warranted to contextualise preferences for contraceptive service delivery among people experiencing social disadvantage. Future work should interrogate inequities between preferred and received modalities of contraceptive service delivery, and explore preferences for service delivery across contraceptive methods necessitating different delivery pathways (e.g., LARC vs. non‐LARC methods).

Our findings underline the importance of a gender‐responsive, intersectionality‐aware approach when researching, designing and delivering inclusive contraceptive services in a digitised healthcare landscape [9]. This work supports recommendations to maintain patient choice in contraceptive service delivery to advance equitable, person‐centred reproductive healthcare [5, 6]. Investment is needed to advance digital health equity by creating an enabling environment whereby all people who choose to can realise the benefits of remote healthcare.

## Author Contributions

O.M., M.P., R.S.F., S.M. and S.P. all contributed to the design of the data collection tool. S.P. developed the concept for this study, with input from S.M. and R.S.F. S.P. conducted the data analysis, with input from N.R. S.P. drafted the manuscript, and all co‐authors reviewed and agreed on the final version.

## Ethics Statement

The Reproductive Health Survey for England (RHSE) was approved by the Observational/Interventions Research Ethics Committee at London School of Hygiene and Tropical Medicine on 25 July 2023 (LSHTM Ethics Ref: 29389). This study was approved by the Faculty of Health and Medicine Research Ethics Committee at Lancaster University on 8th August 2023 (Reference: FHMREC22003 and FHMREC21028).

## Conflicts of Interest

The authors declare no conflicts of interest.

## Supporting information


**Table S1:** Preferred mode of contraceptive service delivery among non‐LARC users.

**Table S2:** Odds ratios for correlates of preference for solely face‐to‐face contraceptive services among study population using non‐LARC methods.

**Table S3:** Preferred mode of contraceptive service delivery among offline sample.

**Table S4:** Characteristics of offline sample, stratified by preference for solely face‐to‐face contraceptive service delivery.

## Data Availability

Data are available on request through the Department of Health and Social Care, via sexualreproductivehealthandabortionpolicyteam@dhsc.gov.uk.
