# Supplementary material for: Preference for Face‐to‐Face Contraceptive Service Delivery Post‐COVID‐19 Pandemic: A Cross‐Sectional Study
Source: BJOG. 2025 Aug 11;132(13):2186–97. doi: 10.1111/1471-0528.18323 (PMC12592788; doi:10.1111/1471-0528.18323)
Supplement: Supplementary file 1 — Table S1: Preferred mode of contraceptive service delivery among non‐LARC users. [file BJO-132-2186-s001.docx]

| **Table S1: Preferred mode of contraceptive service delivery among non-LARC users (n = 17,864)** | |
| --- | --- |
| **Mode of contraceptive service delivery** | **N (%)** |
| Solely face-to-face consultation | 3084 (17.3%) |
| Combination of remote (online/video/telephone) and face-to-face services | 6469 (36.2%) |
| No preference | 3966 (22.2%) |
| Health service website | 2764 (15.5%) |
| Telephone contraceptive consultation | 1330 (7.4%) |
| Video consultation | 251 (1.4%) |
| *LARC: Long-acting reversible contraceptive* | |
