# Supplementary material for: Preference for Face‐to‐Face Contraceptive Service Delivery Post‐COVID‐19 Pandemic: A Cross‐Sectional Study
Source: BJOG. 2025 Aug 11;132(13):2186–97. doi: 10.1111/1471-0528.18323 (PMC12592788; doi:10.1111/1471-0528.18323)
Supplement: Supplementary file 2 — Table S2: Odds ratios for correlates of preference for solely face‐to‐face contraceptive services among study population using non‐LARC methods. [file BJO-132-2186-s002.docx]

| **Table S2: Odds ratios for correlates of preference for solely face-to-face contraceptive services among study population using non-LARC methods (n = 17,864)** | | | | |
| --- | --- | --- | --- | --- |
| **Characteristic** | **Number of participants** | **Percentage reporting preference for solely face-to-face** | **Unadjusted OR**  **(95% CI)** | **Adjusted OR**  **(95% CI)** |
| **Age group** |  |  |  |  |
| 30-39 | 5,572 | 12.6% | 1.00 | 1.00 |
| 16-29 | 9,011 | 20.9% | 1.83 (1.66-2.01) | 1.43 (1.29-1.59) |
| 40-55 | 3,281 | 15.3% | 1.26 (1.11-1.42) | 1.25 (1.10-1.41) |
| **Gender identity** |  |  |  |  |
| Woman/girl | 17,214 | 17.2% | 1.00 | 1.00 |
| Man/boy, non-binary, trans, or thinks of themselves ‘in another way’ | 650 | 19.8% | 1.19 (0.98-1.45) | 0.93 (0.75-1.13) |
| **Born in the UK** |  |  |  |  |
| Yes | 16,095 | 17.3% | 1.00 | 1.00 |
| No | 1,769 | 16.8% | 0.97 (0.85-1.10) | 1.14 (0.99-1.31) |
| **Ethnicity** |  |  |  |  |
| White | 16,531 | 17.4% | 1.00 | 1.00 |
| Asian or Asian British | 417 | 13.4% | 0.72 (0.54-0.95) | 0.81 (0.58-1.12) |
| Black, Black British, Caribbean or African | 191 | 14.1% | 0.78 (0.51-1.16) | 0.95 (0.59-1.45) |
| Mixed or multiple ethnic groups | 642 | 17.6% | 1.03 (0.83-1.26) | 1.07 (0.84-1.36) |
| Other ethnic group | 83 | 21.7% | 1.32 (0.76-2.18) | 1.49 (0.76-2.71) |
| **Degree or equivalent qualification** |  |  |  |  |
| Yes | 13,068 | 15.3% | 1.00 | 1.00 |
| No | 4,796 | 22.7% | 1.61 (1.49-1.75) | 1.27 (1.16-1.39) |
| **In paid employment** |  |  |  |  |
| Yes | 14,352 | 15.8% | 1.00 | 1.00 |
| No | 3,512 | 23.1% | 1.59 (1.45-1.74) | 1.29 (1.17-1.43) |
| **Financially managing** |  |  |  |  |
| Living comfortably | 3,618 | 13.9% | 1.00 | 1.00 |
| Doing alright | 8,211 | 17.0% | 1.27 (1.13-1.41) | 1.13 (1.01-1.26) |
| Just getting by | 4,249 | 19.3% | 1.48 (1.31-1.67) | 1.18 (1.04-1.34) |
| Finding it difficult | 1,786 | 20.5% | 1.60 (1.38-1.86) | 1.18 (1.01-1.38) |
| **NHS Region** |  |  |  |  |
| London | 2,977 | 14.8% | 1.00 | 1.00 |
| South East | 2,589 | 18.4% | 1.29 (1.12-1.49) | 1.17 (1.01-1.36) |
| South West | 1,952 | 17.9% | 1.25 (1.07-1.46) | 1.14 (0.97-1.33) |
| East of England | 1,687 | 16.8% | 1.16 (0.98-1.36) | 1.03 (0.87-1.21) |
| Midlands | 2,599 | 18.3% | 1.28 (1.11-1.48) | 1.16 (1.00-1.34) |
| North East and Yorkshire | 2,115 | 17.4% | 1.21 (1.04-1.40) | 1.12 (0.96-1.31) |
| North West | 1,749 | 17.3% | 1.20 (1.02-1.41) | 1.09 (0.93-1.29) |
| Missing | 2,196 | 17.6% | 1.23 (1.06-1.42) | 1.09 (0.93-1.27) |
| **Disability** |  |  |  |  |
| No | 11,477 | 16.3% | 1.00 | 1.00 |
| Yes | 6,387 | 19.0% | 1.19 (1.10-1.29) | 1.03 (0.95 -1.13) |
| **Relationship status** |  |  |  |  |
| In a relationship, living together | 10,394 | 14.2% | 1.00 | 1.00 |
| In a relationship, not living together | 3,715 | 22.1% | 1.70 (1.55-1.87) | 1.34 (1.20-1.48) |
| Not in a relationship | 3,472 | 21.3% | 1.63 (1.48-1.80) | 1.34 (1.21-1.49) |
| Other, PNTA | 283 | 15.5% | 1.09 (0.77-1.50) | 0.98 (0.70-1.36) |
| **Ethnicity*In paid employment** |  |  |  |  |
| White * Yes | 13,321 | 15.8% | - | 1.00 |
| Asian or Asian British*No | 90 | 15.6% | - | 0.75 (0.37-1.43) |
| Black, Black British, Caribbean or African*No | 37 | 8.1% | - | 0.30 (0.07-0.94) |
| Mixed or multiple ethnic groups*No | 150 | 19.3% | - | 0.72 (0.44-1.15) |
| Other ethnic group*No | 25 | 20% | - | 0.65 (0.19-2.02) |
| *UK: United Kingdom; PNTA: prefer not to answer* | | | | |
