# Supplementary material for: Preference for Face‐to‐Face Contraceptive Service Delivery Post‐COVID‐19 Pandemic: A Cross‐Sectional Study
Source: BJOG. 2025 Aug 11;132(13):2186–97. doi: 10.1111/1471-0528.18323 (PMC12592788; doi:10.1111/1471-0528.18323)
Supplement: Supplementary file 3 — Table S3: Preferred mode of contraceptive service delivery among offline sample. [file BJO-132-2186-s004.docx]

| **Table S3: Preferred mode of contraceptive service delivery among offline sample (n=76)** | |
| --- | --- |
| **Mode of contraceptive service delivery** | **N (%)** |
| Solely face-to-face consultation | 36 (47.4%) |
| Combination of remote (online/video/telephone) and face-to-face services | 28 (36.8%) |
| No preference | 4 (5.3%) |
| Health service website | <5 (<5%) |
| Telephone contraceptive consultation | 7 (9.2%) |
| Video consultation | <5 (<5%) |
