# Supplementary material for: Preference for Face‐to‐Face Contraceptive Service Delivery Post‐COVID‐19 Pandemic: A Cross‐Sectional Study
Source: BJOG. 2025 Aug 11;132(13):2186–97. doi: 10.1111/1471-0528.18323 (PMC12592788; doi:10.1111/1471-0528.18323)
Supplement: Supplementary file 4 — Table S4: Characteristics of offline sample, stratified by preference for solely face‐to‐face contraceptive service delivery. [file BJO-132-2186-s003.docx]

| **Table S4: Characteristics of offline sample, stratified by preference for solely face-to-face contraceptive service delivery (n=76)** | | | | |
| --- | --- | --- | --- | --- |
| **Characteristic** | **All participants** | **No preference for solely face-to-face**  N = 40 (53%) | **Preference for solely face-to-face**  N = 36 (47%) | **P-value** |
| **Age Group** |  |  |  | 0.2 |
| 30 to 39 | 28 (37%) | 11 (28%) | 17 (47%) |  |
| 16 to 29 | 15 (20%) | 8 (20%) | 7 (19%) |  |
| 40 to 55 | 33 (43%) | 21 (53%) | 12 (33%) |  |
| Missing | 0 | 0 | 0 |  |
| **Gender Identity** |  |  |  | >0.9 |
| Woman/girl | 73 (96%) | 38 (95%) | 35 (97%) |  |
| Man/boy, non-binary, trans or thinks of themselves ‘in another way’ | 3 (3.9%) | 2 (5.0%) | 1 (2.8%) |  |
| Missing | 0 | 0 | 0 |  |
| **Born in the UK** |  |  |  | 0.8 |
| Yes | 64 (84%) | 34 (85%) | 30 (83%) |  |
| No | 12 (16%) | 6 (15%) | 6 (17%) |  |
| Missing | 0 | 0 | 0 |  |
| **Ethnicity** |  |  |  | >0.9 |
| White | 61 (80%) | 32 (80%) | 29 (81%) |  |
| Asian or Asian British | 3 (3.9%) | 1 (2.5%) | 2 (5.6%) |  |
| Black, Black British, Caribbean or African | 1 (1.3%) | 1 (2.5%) | 0 (0%) |  |
| Mixed or multiple ethnic groups | 3 (3.9%) | 2 (5.0%) | 1 (2.8%) |  |
| Other ethnic group | 8 (11%) | 4 (10%) | 4 (11%) |  |
| Missing | 0 | 0 | 0 |  |
| **Degree or equivalent qualification** |  |  |  | 0.14 |
| Yes | 32 (42%) | 20 (50%) | 12 (33%) |  |
| No | 44 (58%) | 20 (50%) | 24 (67%) |  |
| Missing | 0 | 0 | 0 |  |
| **In paid employment** |  |  |  | 0.037 |
| Yes | 26 (34%) | 18 (45%) | 8 (22%) |  |
| No | 50 (66%) | 22 (55%) | 28 (78%) |  |
| Missing | 0 | 0 | 0 |  |
| **Self-reported financial hardship** |  |  |  | 0.080 |
| Living comfortably | 9 (12%) | 5 (13%) | 4 (11%) |  |
| Doing alright | 23 (30%) | 17 (43%) | 6 (17%) |  |
| Just getting by | 25 (33%) | 10 (25%) | 15 (42%) |  |
| Finding it difficult | 19 (25%) | 8 (20%) | 11 (31%) |  |
| Missing | 0 | 0 | 0 |  |
| **Index of Multiple Deprivation (IMD) quintile** |  |  |  | 0.3 |
| 5 | 2 (2.6%) | 0 (0%) | 2 (5.6%) |  |
| 4 | 5 (6.6%) | 3 (7.5%) | 2 (5.6%) |  |
| 3 | 6 (7.9%) | 4 (10%) | 2 (5.6%) |  |
| 2 | 13 (17%) | 9 (23%) | 4 (11%) |  |
| 1 | 35 (46%) | 15 (38%) | 20 (56%) |  |
| Missing | 15 (20%) | 9 (23%) | 6 (17%) |  |
| **Self-reported health status** |  |  |  | 0.038 |
| Good | 37 (49%) | 25 (63%) | 12 (33%) |  |
| Fair | 25 (33%) | 10 (25%) | 15 (42%) |  |
| Bad | 14 (18%) | 5 (13%) | 9 (25%) |  |
| Missing | 0 | 0 | 0 |  |
| **Disability** |  |  |  | 0.048 |
| Yes | 46 (61%) | 20 (50%) | 26 (72%) |  |
| No | 30 (39%) | 20 (50%) | 10 (28%) |  |
| Missing | 0 | 0 | 0 |  |
| **Relationship status** |  |  |  | 0.6 |
| In a relationship, living together | 21 (28%) | 12 (30%) | 9 (25%) |  |
| In a relationship, not living together | 16 (21%) | 9 (23%) | 7 (19%) |  |
| Not in a relationship | 37 (49%) | 19 (48%) | 18 (50%) |  |
| Other, PNTA | 2 (2.6%) | 0 (0%) | 2 (5.6%) |  |
| Missing | 0 | 0 | 0 |  |
| IMD: index of multiple deprivation; PNTA: prefer not to answer | | | | |
